# The effect of manual therapy to the thoracic spine on pain-free grip and sympathetic activity in patients with lateral epicondylalgia humeri. A randomized, sample sized planned, placebo-controlled, patient-blinded monocentric trial

**DOI:** 10.1186/s12891-020-3175-y

**Published:** 2020-03-24

**Authors:** Philipp Zunke, Alexander Auffarth, Wolfgang Hitzl, Mohamed Moursy

**Affiliations:** 1 Physiozentrum Salzburg, Innsbrucker Bundesstraße 35, 5020 Salzburg, Austria; 2 Department of Orthopedics and Traumatology, Paracelsus Medical University Salzburg, Muellner Hauptstr. 48, 5020 Salzburg, Austria; 3 Paracelsus Medical University Salzburg, Research Office (biostatistics), Strubergasse 20, 5020 Salzburg, Austria; 4 Department of Ophthalmology and Optometry, Paracelsus Medical University Salzburg, Muellner Hauptstr. 48, 5020 Salzburg, Austria; 5 Research Program Experimental Ophthalmology and Glaucoma Research, Paracelsus Medical University, Muellner Hauptstrasse 48, 5020 Salzburg, Austria

**Keywords:** Lateral epicondylalgia, Tennis elbow, Thoracic spine, Manual therapy, Sympathetic activity, musculoskeletal pain

## Abstract

**Background:**

The treatment of first choice for lateral epicondylalgia humeri is conservative therapy. Recent findings indicate that spinal manual therapy is effective in the treatment of lateral epicondylalgia. We hypothesized that thoracic spinal mobilization in patients with epicondylalgia would have a positive short–term effect on pain and sympathetic activity.

**Methods:**

Thirty patients (all analyzed) with clinically diagnosed (physical examination) lateral epicondylalgia were enrolled in this randomized, sample size planned, placebo-controlled, patient-blinded, monocentric trial. Pain-free grip, skin conductance and peripheral skin temperature were measured before and after the intervention. The treatment group (15 patients) received a one-time 2-min T5 costovertebral mobilization (2 Hz), and the placebo group (15 patients) received a 2-min one-time sham ultrasound therapy.

**Results:**

Mobilization at the thoracic spine resulted in significantly increased strength of pain-free grip + 4.6 kg ± 6.10 (*p* = 0.008) and skin conductance + 0.76 μS ± 0.73 (*p* = 0.000004) as well as a decrease in peripheral skin temperature by − 0.80 °C ± 0.35 (*p* < 0.0000001) within the treatment group.

**Conclusion:**

A thoracic costovertebral T5 mobilization at a frequency of 2 Hz shows an immediate positive effect on pain-free grip and sympathetic activity in patients with lateral epicondylalgia.

**Clinical trial registration:**

German clinical trial register DRKS00013964, retrospectively registered on 2.2.2018.

## Background

Lateral epicondylalgia, colloquially called tennis elbow, is a common musculoskeletal disorder within the working population between 35 and 55 years of age [1–3]. It affects 40% of the population once in a lifetime [4] at a prevalence of 1–3% [1]. This pathology involves the tendons that connect the forearm extensors to the lateral epicondyle of the humerus [2]. In this respect, the extensor carpi radialis brevis of the dominant arm is involved in most cases [3, 5]. Pain is typically located around the lateral epicondyle of the humerus and is intensified by extension of the elbow in combination with wrist flexion. Resisted wrist extension, extension of the second and third fingers, as well as gripping also cause pain [6–8].

The treatment of first choice for lateral epicondylalgia is conservative therapy. Most patients will recover without surgery within 1 year after the first occurrence [9]. A variety of conservative treatment strategies positively influence the course of this pathology [7]. Especially treatment strategies like eccentric exercise, stretching or manual therapy have been reported to be effective [10–12]. Apart from these, Vicenzino, et al. [13] and Fernandez-Carnero, et al. [14] supported the assumption that spinal manual therapy (SMT) of the cervical spine would have a positive short-term effect on pain-free grip and the threshold of pain provoked by pressure to the lateral humeral epicondyle. Furthermore, there is an indication that SMT applied to the thoracic spine also has a positive short-term effect on pain-free grip (PFG) [14]. Thus, we hypothesize that SMT applied to the cervical or thoracic spine activates mechanisms that cannot be explained through local reactions only [15].

Bialosky, et al. [16] proposed in their comprehensive model that manual techniques are effective not only due to mechanical, neurophysiological, peripheral and spinal mechanisms but also induce supraspinal pain inhibition associated with sympathoexcitation This effect of hypoalgesia occurred rapidly after SMT [17]. Such a sympathetic response was indirectly and contemporaneously recorded by measuring skin temperature (TEMP), skin conductance (SC), cortisol levels and heart rate [16]. Skin temperature decreased as a result of vasoconstriction caused by activation of sympathetic fibers [18]. Skin conductance increased after a sympathetic sudomotor activation in connection with sweat production [19]. Kingston et al. assumed that this reaction was implemented by the dorsal periaqueductal gray [20], which was first described by Reynolds [21]. The dorsal periaqueductal gray is, along with the ventrolateral region of the medulla, parabrachial nuclei and the hypothalamus, an important part in the central autonomic nervous system [22]. It plays a major role in the body’s own descending pain inhibitory system and is part of the midbrain’s periaqueductal gray [23, 24]. The dorsal periaqueductal gray facilitates short-lasting, non-opioid mediated analgesia, hyperventilation and sympathoexcitation in the context of the fight-or-flight reaction [25–27]. The peripheral sympathetic nervous system, the sympathetic trunk, is located at the thoracic spine between the first thoracic (T1) and the second lumbar (L2) vertebrae. Thus, the upper thoracic spine connects sympathetic reactions to the upper extremities and the lower thoracic spine to the lower extremities [22, 28, 29]. Additionally it might be worth to focus on the SNS in tendinopathy as a recent review suggests that there is be an increased activity in paratendinous tissue of painful tendons [30] and might be associated with the pain duration as shown in Achilles tendinopathy [31].

So far, sympathoexcitation after SMT has been demonstrated in asymptomatic populations, as Petersen, et al. [32] showed an increase in skin conductance following a grade III oscillatory technique at the fifth cervical vertebra (C5) compared to the placebo and control group. Moulson and Watson [33] presented an analog result where sympathetic nervous system (SNS) activity rose after Mulligan’s sustained natural apophyseal glides (SNAGs) at the C5/6 joint [34]. Chiu and Wright [35] showed that skin conductance increased significantly after a 2 Hz (120/min mobilization rate) posterior-anterior mobilization at C5/6 compared to a 0.5 Hz mobilization in their control group [36]. Jowsey, et al. [25] investigated whether a 0.5 Hz thoracic mobilization would have a greater effect on skin conductance than a placebo intervention. Their mobilization group provided preliminary evidence that a mobilization of T4 can produce sympathoexcitatory effects in the hands, which was measured 5 min after the mobilization [25]. Tsirakis and Perry [37] provided preliminary evidence that modified Mulligans’s spinal mobilization with a leg motion technique evoked a side-specific sympathetic change in healthy subjects within the treatment group.

Some research indicates that there is also a sympathetic response after SMT in patient with musculoskeletal pain. There is a preliminary indication for an sympathoexcitation for patients with mechanical, unspecific neck pain [38–40], epicondylitis lateralis humeri [41] or also low back pain [42].

In summary, there is evidence that SMT enforces measurable SNS activity in an asymptomatic population. Because there is little of evidence for such reactions among a symptomatic population [20, 43], this study is a further step to fill this gap.

We hypothesize that a grade III spinal manual therapy directed to the ribs of T5 on the affected side with 2 Hz increases pain-free grip and excites peripheral sympathetic activity correlating with skin conductance increase and a skin temperature decrease in patients with lateral epicondylalgia humeri.

## Methods

The ethics commission in Salzburg/ Austria approved this research project with the official notice labelled 415-E/2158/4–2017. Written informed consent was obtained from all participants.

### Research design

Randomized, sample size planned, patient-blinded, placebo-controlled trial, monocentric, independent group design, intention-to-treat, trial.

### Participants

Patients were diagnosed and recruited at the elbow department - University Clinic of Orthopedics and Traumatology in Salzburg between May 2017 and December 2017. Women and men aged between 18 and 55 years with unilateral, acute and subacute (pain duration did not exceed 6 month) lateral epicondylalgia humeri were included. The clinical examination was carried out by an elbow expert, included following: inspection, palpation, range-of-movement, peripheral blood circulation, sensibility, motor activity, nerve bottleneck. Provocation tests for epicondylitis, one was required to be positive, were also included according to Vicenzino et al. [41]: gripping, resisted contraction wrist extensors with m. carpi radialis brevis, stretching the forearm extensors or pain on the lateral epicondyle during palpation. Imaging was not used as it is not recommended for non-chronic elbow pain [2, 7, 44]. However, patients with unclear clinical presentation or possible differential diagnosis were not eligible for randomization. Common differential diagnosis for lateral epicondylalgia are: cervical radiculopathy, posterolateral rotation instability, radial tunnel syndrome, plica syndrom, radio- capitellar arthrosis or osteochondritis dissecans of the capitellum [45, 46]. The clinical examination was based on the AWMF Guideline 033–019 Epicondylopathia radialis humeri [44].

Additional exclusion criteria were predefined and retrieved anamnestic from patients’ case histories: bilateral elbow pain, osteoporosis, tumor diseases, history of operations on the elbow or thoracic spine, acute thoracic pain, pregnancy, oral anticoagulation, central pain medication, and biased to manual therapy. Patients were asked to avoid stimulating substances like caffeine before testing [47].

Thirty patients (17 females and 13 males) at a mean age of 45.1 ± 8.5 years met the inclusion criteria and gave written consent to participate (Table 1).

**Table 1:**
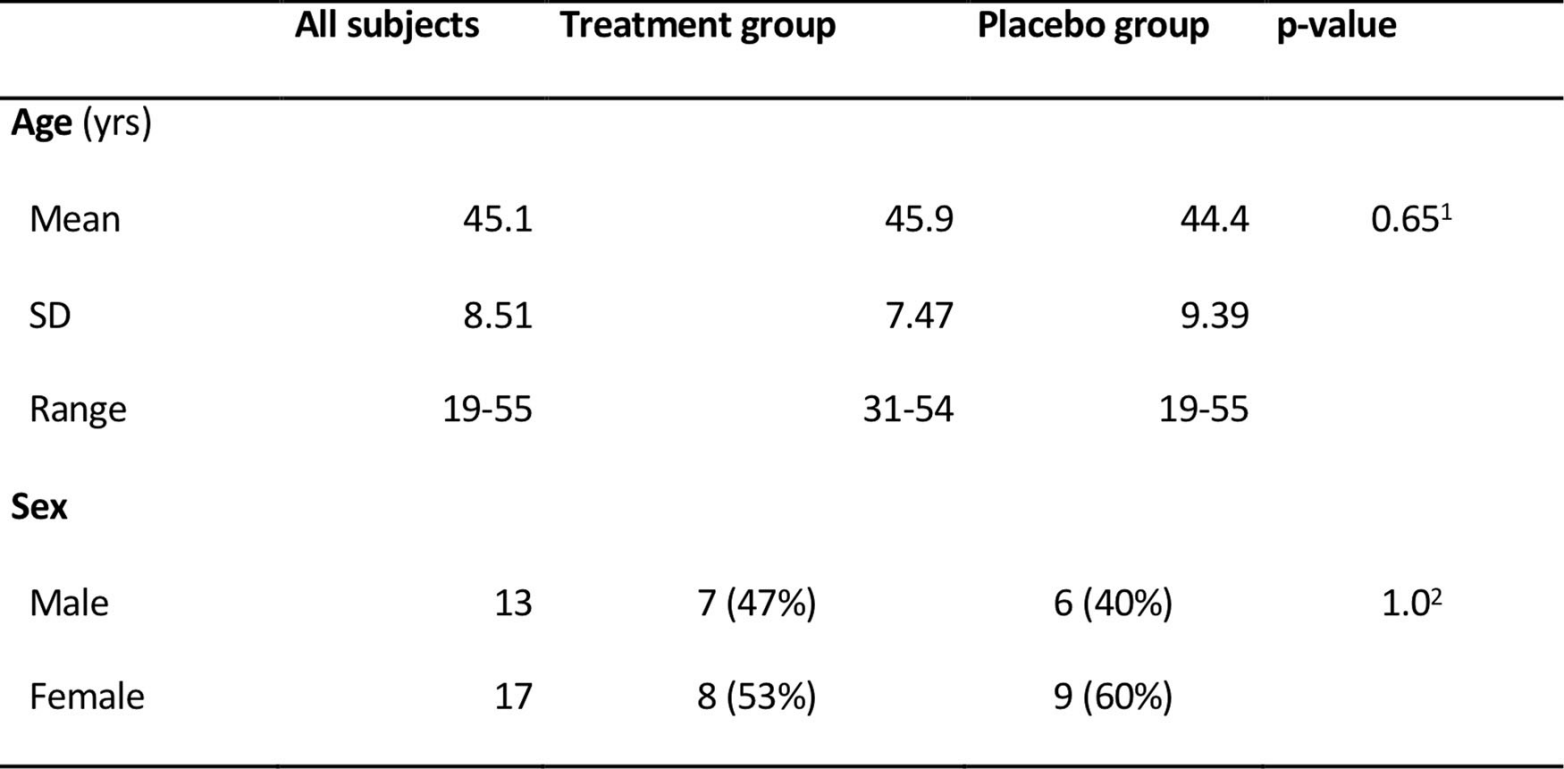
Patient demographics (^*1*^*)Two-sided, independent t-test,*^*2*^*)Two-sided, Fisher’s Exact test*)

### Study setup

The temperature of the examination room was maintained at a constant 22 °C and sound insulated. Patients gave signed consent and were randomized into two groups and blinded to which group they were assigned. The patients laid supine and performed a PFG test, which is a highly reliable and valid test for the examined pathology [48, 49]. The test was performed on the non-affected and affected side. It was carried out in a supine position with the arms next to the body, the elbow extended and a prone hand. For the PFG test, patients gripped a manometer as strongly as possible on the non-affected side and only until pain occurred on the affected side.

**Fig. 1:**
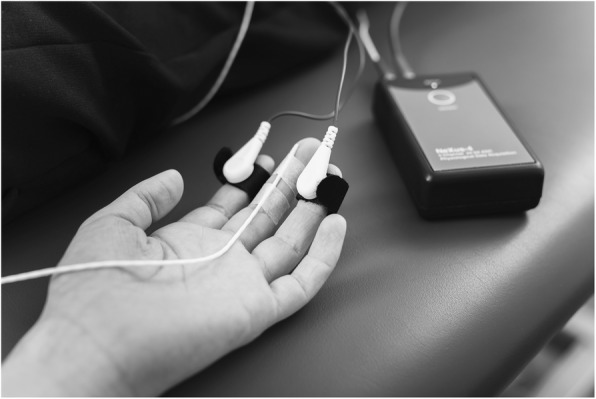
Measurement points for PGF, SC and TEMP at baseline and after intervention

**Fig. 2:**
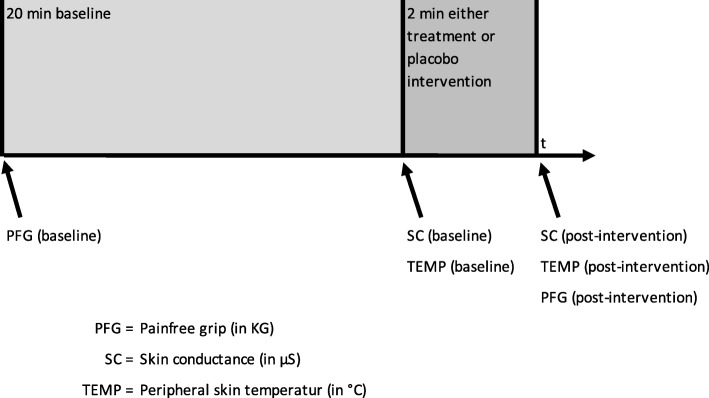
Electrode positioning for SC (II+IV finger) and TEMP (III finger)

To measure SC, patients were placed in a prone position and the researcher identified the spinous process of T5 by counting downwards from the vertebra prominens (C7) and labeled it. The subjects were placed with the cervical spine in neutral rotation with the arms next to the body and palms up. Fingertips and sensors were cleaned with a disinfectant containing 73.5% ethanol, and they were given sufficient time to dry before attaching the electrodes. Sensors were placed on the tip of the index and ring fingers, and the TEMP sensor was fixed to the palmar surface of the middle finger (Fig. 1). Subjects underwent a 20-min stabilization period [50] followed by either the treatment or placebo, each for two minutes (Fig. 2). Patients were asked to breathe calmly and refrain from conversation during data collection. The intention of this stabilization period was to reach a baseline level of relaxation. SC and TEMP data were collected simultaneously during the last 10 s of this stabilization period (baseline) and during the last 10 s of the following treatment or placebo intervention. After the intervention patients were positioned supine as described in the pre-interventions protocol. Patients gripped the manometer again as strongly as possible on the non-affected side and then, only until pain occurred, on the affected side.

### Treatment intervention

**Fig. 3:**
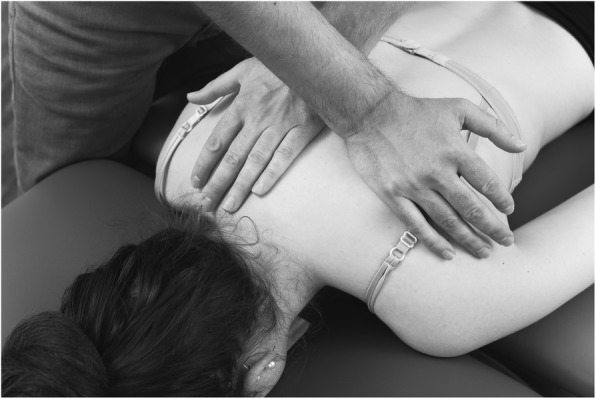
Costo-vertebral joint mobilization

A grade III mobilization, which is defined as a large-amplitude oscillating mobilization until the end of movement [36] of the ribs at T5, was performed at 2 Hz (120 impulses per minute) for two minutes. The researcher stood contralateral to the affected side next to the subject. The transverse processes of the contralateral side from T4-T6 were stabilized while the rib at T5 on the affected side was mobilized (Fig. 3). The direction of the mobilization was posterior-anterior and lateral and cranio-caudal according to Jowsey, et al. [25]. This specific technique was chosen due to the anatomical positioning of the sympathetic trunk dorsal to the costovertebral joints [51].

### Placebo intervention

A sham ultrasound therapy (ultrasound gel and transducer with room temperature) was performed on the same segment as in the treatment group for 2 min. The setting and procedure were identical to those in the treatment group. Care was taken to ensure that no pressure was applied to the costovertebral joints in order to avoid a mechanical stimulus.

### Technical information

PFG (in kg) was measured with a calibrated digital hand dynamometer, model KERN MAP 80K1S, Kern & Sohn GmbH, 72,336 Balingen-Frommern, Germany. Skin conductance (sampling rate: 32 SPS in microsiemens (μS)) and skin temperature (32 SPS in degree celsius (°C)) were measured with a biofeedback Nexus-4 device and BioTrace+ Software V2015B1, Mind Media B.V., 6049 CD Herten, the Netherlands.

### Statistical analysis

Sample size calculation: The importance of a sample size computation in this research area was pointed out by Farrokhyar, et al. [52]. PFG was used as the primary outcome measurement, and the standard deviations used for this computation are based on results in the thoracic mobilization group out of the study from Fernandez-Carnero, et al. [14] which are s1 = 3.7 in group 1 and s2 = 1 in group 2. To detect a difference of 3 units with 90% power and a nominal alpha level of 5%, a total of 30 patients with clinically diagnosed lateral epicondylalgia humeri were randomized into two groups with 15 patients each. Sample size computation was done using PASS 13 [53].

Randomization: Based on the sample size planning, a randomization list was generated by using computer-generated random numbers and based on the random sorting method [54].

Blinding: Patients were blinded to the allocation of the group. They did not know which group was the assumed efficient or the placebo treatment.

Data evaluation methods: Data consistency was checked, and data were screened for outliers and normality using quantile plots. Continuous variables were also tested for normality using the Kolmogorov-Smirnov test. Cross tabulation tables were used and analyzed with Fisher’s Exact test. After carefully testing all assumptions, a repeated measures ANOVA was done to test for group, time and interaction effects. Least significant different tests as post hoc tests were performed as one-sided as described in the sample size computation. The 95% confidence intervals for means were computed, and Whisker plots were used to illustrate the results. A *p*-value < 0.05 was considered statistically significant. All statistical analyses in this report were performed by use of NCSS [55] and STATISTICA 13 [56].

## Results

All 30 included subjects were randomized, tested and analyzed. The dropout rate was 0%. There were no immediate adverse effects or complications recorded. Long term adverse effects were not reported by the subjects themselves and not queried by the researchers as a follow-up was not planned.

### Pain-free grip on the non-affected side

**Fig. 4:**
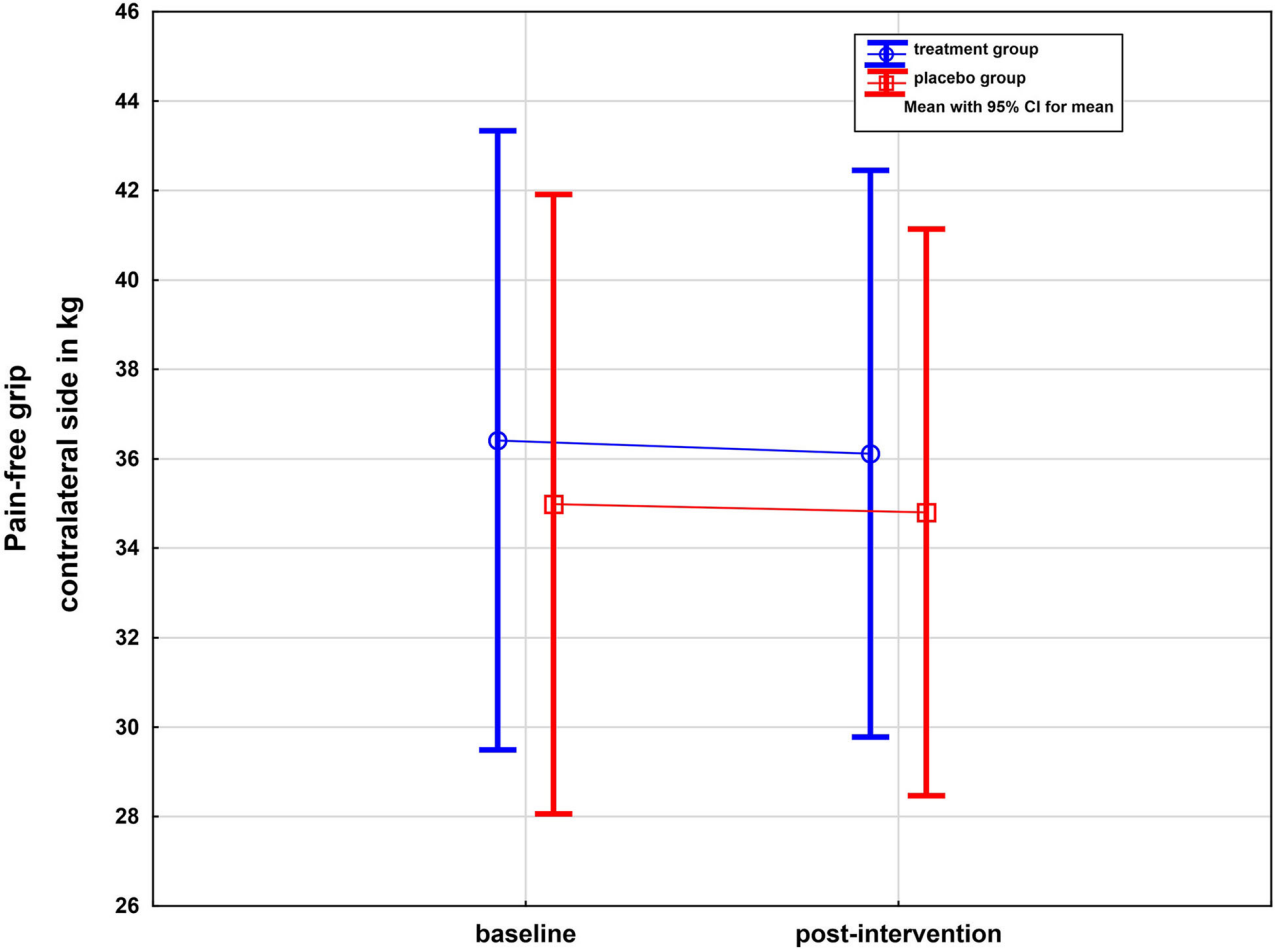
Whisker plot of PFG of the unaffected side in KG in the treatment and placebo groups: baseline and post-intervention

There was no detectable change in PFG on the non-affected side, either in the treatment or in the placebo group. The repeated measures ANOVA reported no significant effects (*p* = 0.77, one-sided) (Table 2 and Fig. 4). Generally, there was a trend of greater strength at baseline and after intervention in the treatment group.

**Table 2:**
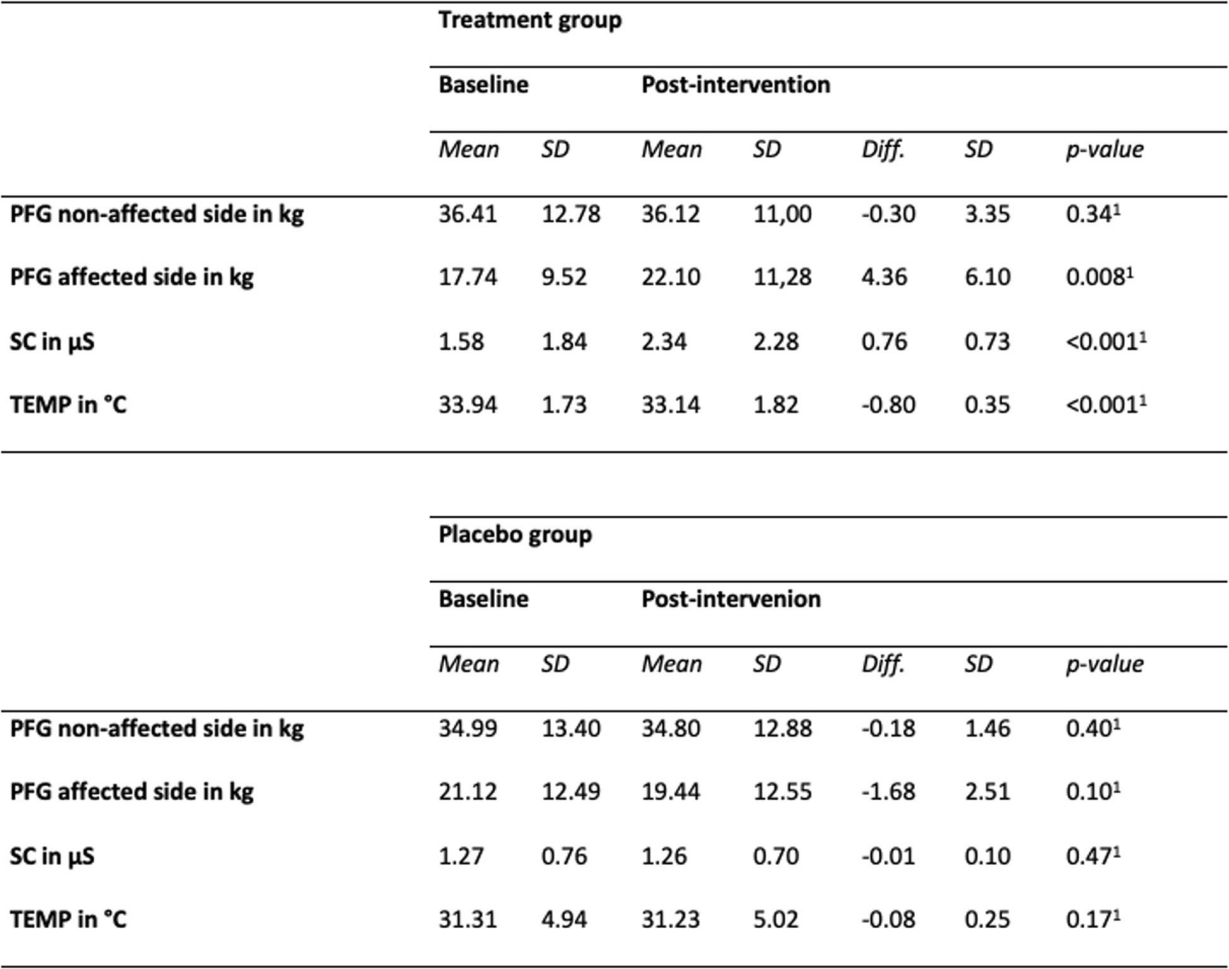
Baseline and post-intervention measurements for PFG, SC and TEMP (^*1*^*)one-sided*)

### Pain-free grip on the affected side

**Fig. 5:**
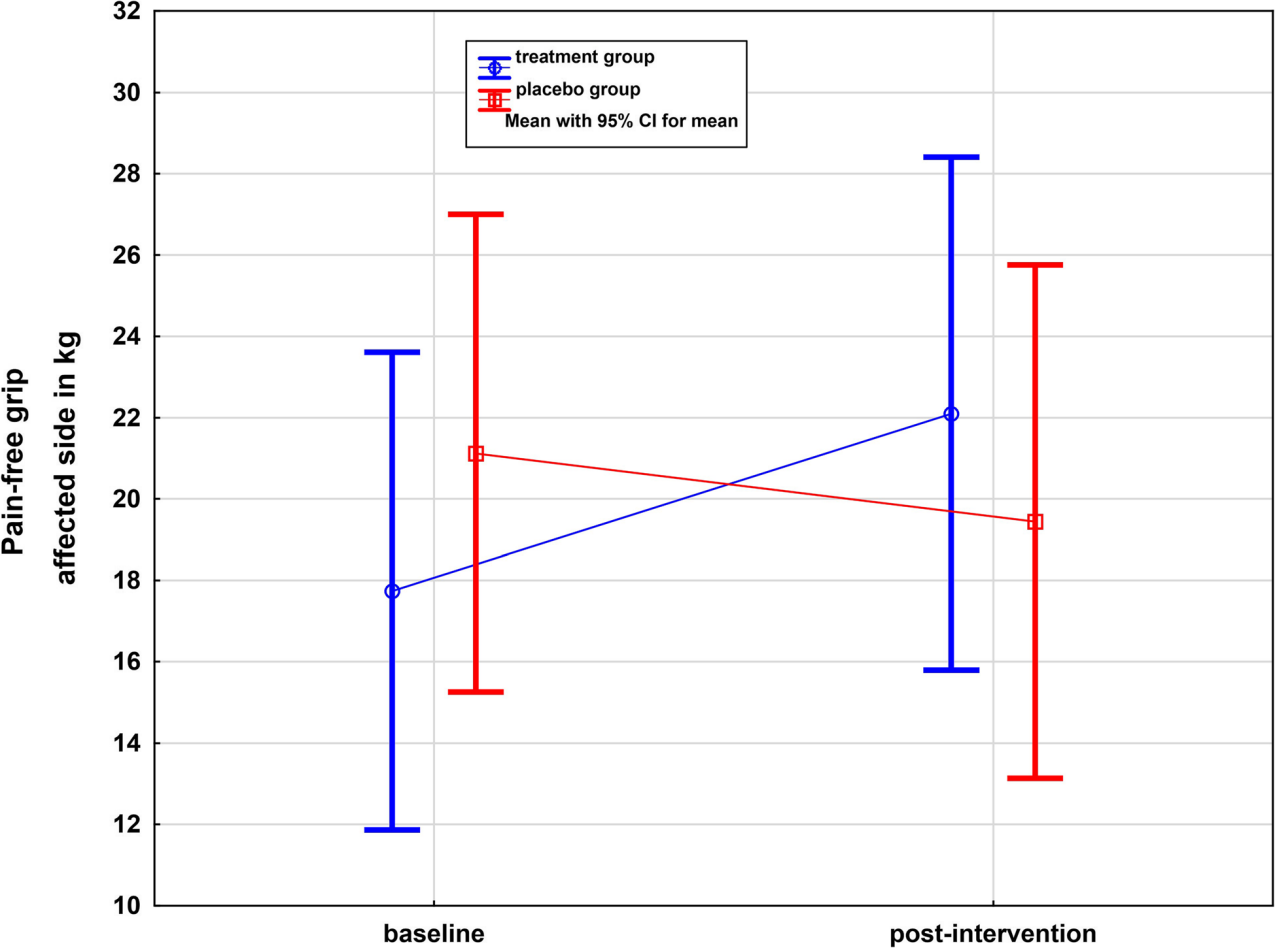
Whisker plot of PFG of the affected side in KG in treatment and placebo groups: baseline and post-intervention. Significant difference over time in the treatment group (*p* = 0.008, one-sided)

Within the treatment group, PFG significantly increased by 4.36 kg (95% CI: 1.8–6.92) from 17.74 kg to 22.10 kg (24.6%), which corresponds to an increase of side over time in the treatment group (*p* = 0.008, one-sided) (Table 2 and Fig. 5). In contrast, no significant change was detected in the placebo group (*p* = 0.10, one-sided).

### SC differences on the affected side

**Fig. 6:**
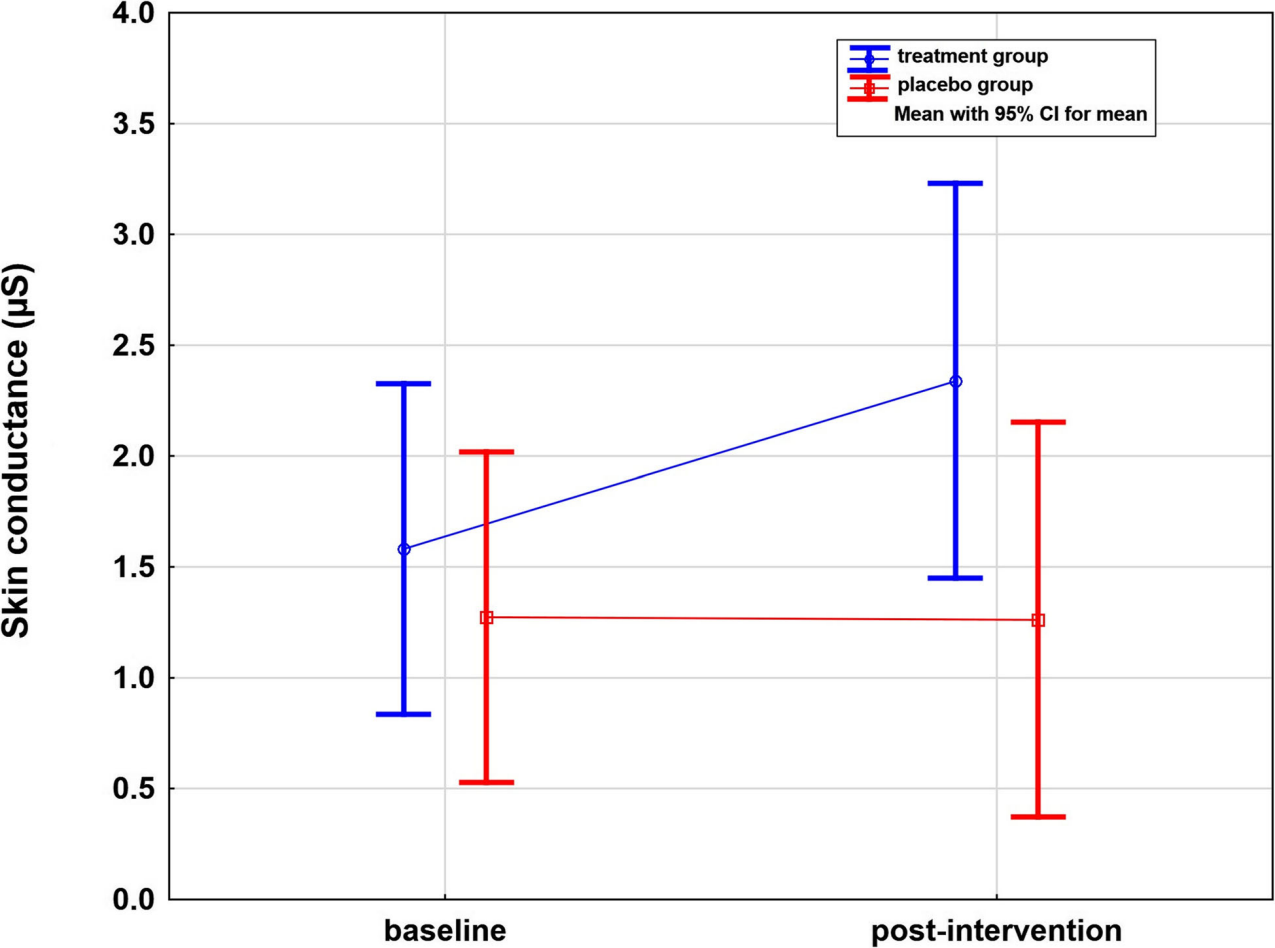
Whisker plot of skin conductance of the affected side in μS in the treatment and placebo groups: baseline and post-intervention. Significant difference over time in the treatment group (*p* < 0.001, one-sided). Significant difference between groups in post-intervention (*p* = 0.033, one-sided)

The SC significantly increased by + 0.76 μS ± 0.73 (48%) from 1.58 μS at baseline to 2.34 μS after intervention on the affected side in the treatment group (*p* < 0.001, one-sided). There was no such change detectable in the placebo group (*p* = 0.47, one-sided). A significantly (*p* = 0.033, one-sided) greater SC was recorded for the treatment group comparing measurements at baseline and after intervention (Table 2 and Fig. 6).

### Temperature differences on the affected side

**Fig. 7:**
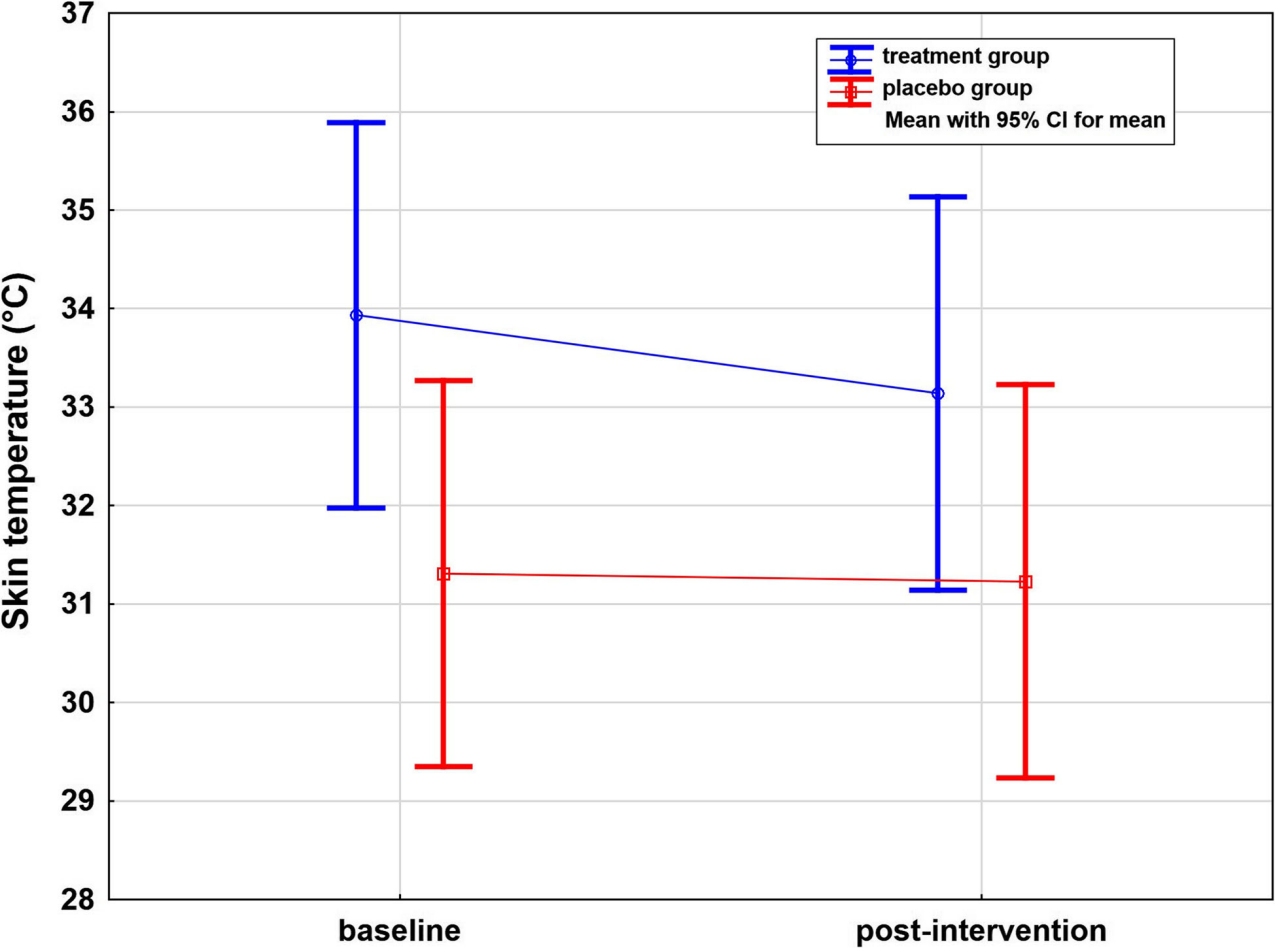
Whisker plot of skin temperature of the affected side in °C in the treatment and placebo groups: baseline and post-intervention. Significant difference over time in the treatment group (*p* < 0.001, one-sided)

The TEMP significantly decreased by 0.80 °C ± 0.35 (2.4%) from 33.94 °C to 33.14 °C on the affected side after treatment (*p* < 0.001, one-sided). There was no such change in the placebo group (*p* = 0.17, one-sided). No significant difference between the treatment and placebo groups was found after treatment (Table 2 and Fig. 7).

## Discussion

This study investigates the short-term effects of thoracic mobilization on PFG and SNS activity in patients with lateral epicondylalgia.

There are consistent findings concerning pain-free grip changes in patients with lateral epicondylalgia. Fernandez-Carnero, et al. [14] detected a 19.8% PFG increase within the thoracic manipulation group and an increase of 24.7% with cervical mobilization. Still, it seems that thoracic manipulation is equally effective as they found no statistically significant difference between the groups. Other trials showed an initial effect on PFG after a cervical spine manipulative physiotherapy treatment (+ 13.98 N ± 5.26) and a cervical lateral glide mobilization (+ 33.2 N) [13, 41].

Vicenzino, et al. [41] recorded a skin conductance change of 69% in the hand after the cervical mobilization in patients with lateral epicondylalgia. There are similar outcomes for SNS activity after mobilizations in other patient populations. Sterling, et al. [39] provide evidence that skin conductance increases by 16% after a grade III posterior-anterior technique on the articular pillar of C5/6 compared with the placebo and control groups in patients with mid- to lower cervical pain over 3 months and a dysfunction of C5/6. Additionally, skin temperature decreased by 2.5 ± 0.5%. Lascurain-Aguirrebena, et al. [38] demonstrated an effective reduction of symptoms and an immediate rise in sympathetic electrodermal activity during a grade II-III unilateral cervical SMT on patients with non-specific neck pain. Perry, et al. [42] showed that a lumbar rotatory manipulation significantly increases skin conductance (+ 255 ± 141%) in the foot during the treatment of patients with low back pain. Furthermore, an increase in SNS activity after spinal mobilizations was found for asymptomatic patients [25, 32, 35, 57]. The findings outlined above are comparable to the 48.1% increase in SC after thoracic mobilization in our study.

Other authors suspected a connection between pain at the lateral elbow and pain in the cervical or thoracic spine regarding lateral epicondylalgia as not just a localized pathology [6, 58]. Such coexistence of thoracic dysfunction was also found with other musculoskeletal disorders like neck or low back pain [1]. Some assume that restricted motion in the cervicothoracic spine may lead to shoulder girdle dysfunctions and shoulder pain [59, 60]. This connection in the so-called regional interdependence model [61] might also be a supplementary indicator that reactions after SMT cannot be explained by local reactions alone but may be explained by non-specific reactions and neurophysiological mechanisms [60]. Recent studies have shown an immediate positive effect after a thoracic mobilization for mechanical cervical pain and shoulder impingement [62–64].

According to the improvement of peripheral outcome measures, our finding leads to the conclusion that thoracic spinal mobilization activates the body’s own descending pain inhibitory mechanisms in patients with lateral epicondylalgia. This effect goes beyond local mechanisms of pain inhibition and is widely discussed in the current literature [15, 16, 25, 57]. Practitioners should be aware of the body’s own pain inhibitory mechanisms. Conservative therapies for lateral epicondylalgia should not only focus in the elbow and the close surrounding structures. We only measured short term effects; future studies should consider evaluating whether patients with lateral epicondylalgia radialis benefit from a mobilization to the thoracic spine in a long-term study. It also seems to be interesting if subgroups with thoracic joint restrictions have an impact on the effect [6, 58]. Furthermore, studies that deal with symptomatic populations are underrepresented in the current literature [20, 43]. It is not clear whether the positive results can be transferred to different symptomatic patient populations.

There are some limitations to this study. First, we only investigated the immediate effect after a thoracic mobilization and abstained from collecting SNS follow-up data in favor of collecting pain-free grip data as soon as possible after the intervention. We are aware that skin conductance and skin temperature are some kind of notorious for their liability and that there are also other measurement parameters like heart rate variability or salivary cortisol levels. Regardless that fact we decided for the parameters used as they are easy to access and also might be side specific [25]. Second, the thoracic spine was not examined for any joint restrictions. It is known that positive responses for provocation tests in the cervical and thoracic spine are more common in patients with lateral elbow pain than in a healthy control population [58]. The duration of lateral epicondylalgia was not surveyed, though we were aware that there might be a local and central sensitization and spread of pain mechanisms in chronic disorders [65]. Furthermore, we didn’t collect data of possible psychosomatic factors that might affect the outcomes [66].

The strength of our study is the planned sample size, randomization and patient blinding. Sample size calculation is important to detect differences with appropriate power [67]. We were able to demonstrate a significant difference with 90% power due to the planned sample size. Randomization ascertains comparable groups and thereby eliminates possible bias. Patients were blinded to their treatment, which functioned to minimize the expectation bias.

## Conclusion

Thoracic costovertebral T5 mobilization at a frequency of 2 Hz has immediate unilateral positive effects recorded as an increase in pain-free grip and sympathetic activity in patients with lateral epicondylalgia. Because this is the first study on thoracic mobilization in a population with lateral epicondylalgia, there is a need for further investigation. As cervical and thoracic SMT seem effective, they are of interest, as they could have a greater impact on pain and SNS activity. Furthermore, the long-term effects of SMT techniques have to be included in future investigations.

## Data Availability

The datasets used and/or analysed during the current study are available from the corresponding author on reasonable request.

## Abbreviations

°C: Degree celsius
C: Cervical
L: Lumbar
PFG: Pain-free grip
SC: Skin conductance
SMT: Spinal manual therapy
SNAG: Sustained natural apophyseal glides
SNS: Sympathetic nervous system
T: Thoracic
TEMP: Skin temperature
μS: microsiemens
